# Effects of Ethnic Classification on Substantive Findings in Adolescent Mental Health Outcomes

**DOI:** 10.1007/s10964-022-01612-6

**Published:** 2022-04-19

**Authors:** Esther S. Yao, Pat Bullen, Kane Meissel, Jemaima Tiatia, Theresa Fleming, Terryann C. Clark

**Affiliations:** 1 Faculty of Education and Social Work, The University of Auckland, Auckland, New Zealand; 2 School of Māori Studies and Pacific Studies, The University of Auckland, Auckland, New Zealand; 3 School of Health, Victoria University of Wellington, Wellington, New Zealand; 4 School of Nursing, The University of Auckland, Auckland, New Zealand

**Keywords:** Adolescence, Mental health, Race/ethnicity, Multiple ethnicities, Ethnic classification, Methods

## Abstract

Although most adolescents are healthy, epidemiological studies show that a significant number experience mental health challenges, and that Indigenous and ethnic minority youth tend to have poorer mental health outcomes. However, ethnic classification in adolescence is complex due to increasing multi-ethnic identification, and little is known about how different classification methods affect research conclusions. This study used a nationally representative adolescent sample from Aotearoa New Zealand (*N* = 8275; ages 12–18; 55% female; 32% multi-ethnic) to investigate the effects that five ethnic classification methods have on substantive findings in three mental health outcomes: overall psychosocial difficulties, deliberate self-harm, and suicide attempts. The results showed that, depending on the classification method used, reported outcomes within the same nominal ethnic group varied by an effect size (*d*) of up to 0.12, and the reported magnitude of difference between nominal ethnic groups varied by an effect size (*d*) of up to 0.25. These effects are substantial given that they are solely due to a change in method. The impact that ethnic classification method has on substantive findings highlights the importance of criticality and transparency in research involving ethnicity data.

## Introduction

While most adolescents are healthy, mental health disorders affect around one in five young people and are one of the leading causes of morbidity and disability in adolescence (Thapar et al., 2012). Concerningly, epidemiological studies show that mental health challenges and associated indicators of distress, such as deliberate self-harm (i.e., non-suicidal self-injury) and suicide attempts, tend to disproportionately affect youth from Indigenous and ethnic minority backgrounds (Anderson & Mayes, 2010). However, classifying ethnicity for research in multi-ethnic contexts is complex. Previous studies, primarily conducted with adult physical health outcomes, suggest that researchers’ choice of ethnic classification method can result in different substantive findings, and hence alter the implications and conclusions drawn (e.g., Mays et al., 2003; Ministry of Health, 2008). However, little is known about how the choice of ethnic classification method affects results in the field of adolescent mental health. Therefore, the current study uses a nationally representative adolescent sample from the multi-ethnic country of Aotearoa New Zealand to examine how different ways of classifying self-identified ethnicity affect substantive findings in the analysis of three mental health outcomes: overall psychosocial difficulties, deliberate self-harm, and suicide attempt.

### Ethnic Differences in Adolescent Mental Health Outcomes

Ethnicity—defined as socially-constructed groups with shared ancestry, history, traditions, culture, values, and beliefs (Morning, 2008)^1^—is a variable that has often been found to be associated with adolescent mental health outcomes, even after accounting for demographic characteristics such as socioeconomic deprivation. For example, a comprehensive review into internalising disorders in the United States indicates that racial/ethnic minority youth, particularly Hispanic youth, tend to have significantly poorer mental health outcomes when compared to non-Hispanic Whites (Anderson & Mayes, 2010). In Aotearoa New Zealand, a nationally representative survey series of secondary school students show that, in comparison to New Zealand European youth, Māori (Indigenous Peoples) and Pacific youth (those originating from neighbouring Pacific nations, e.g., Sāmoa, Cook Islands, and Tonga) tend to have significantly poorer mental health outcomes, including higher levels of mental health distress (Fleming et al., 2020a), self-harm (Fortune et al., 2010), and suicide attempts (Clark et al., 2018). The literature highlights racism at both the individual level (e.g., prejudice and discrimination based on race/ethnicity; Benner et al., 2018) and institutional level (e.g., unequal distribution of opportunities and resources; Williams, 2018) as major contributing factors. These mental health inequities are concerning from both developmental and equity perspectives alike.

Consequently, ethnicity is a variable that is crucial for monitoring, understanding, and addressing adolescent mental health concerns. First, ethnicity data are needed to monitor whether ethnic inequities are improving or worsening over time; for example, via trends in prevalence rates (Fleming et al., 2020a). Second, ethnicity data are needed to understand the factors underlying ethnic disparities; for example, by testing models of risk and protective factors associated with each ethnic group (Adkins et al., 2009). Third, ethnicity information is needed to target and evaluate interventions and policies aimed at decreasing ethnic inequities; for instance, by assessing whether an intervention is equally effective for each targeted ethnic group, or if it is underserving a particular group (Mays et al., 2003).

Regression analysis is a statistical technique commonly used for ethnicity-based mental health analyses. A basic regression model usually specifies a mental health outcome as the dependent variable, and ethnicity and other relevant demographic characteristics as independent variables (Clark et al., 2018). These characteristics include: age, as there is typically a post-pubertal rise in mental health challenges due to biological, cognitive, and social changes (Thapar et al., 2012); sex, as adolescent females are around two times more likely to report mental health distress (Thapar et al., 2012); and the contextual characteristics of socioeconomic deprivation and urbanicity, as lower income and/or urban areas have been associated with poorer mental health outcomes (Fleming et al., 2020a; Kessler et al., 2012). More complex models may incorporate additional independent variables, such as experiences of racism and discrimination, to seek to understand reasons underlying ethnic differences (Crengle et al., 2012).

However, many of these variables are social constructs (e.g., ethnicity, socioeconomic deprivation, urbanicity), so the way they are operationalised can influence results (Gillborn et al., 2018). This is particularly pertinent to ethnicity, as due to migration, interethnic unions, and changing patterns of self-identification, an increasing proportion of young people identify with more than one ethnic group (Aspinall, 2018). This raises the methodological issue of how to categorise multiple ethnic identifications for statistical analysis, and the question of how researchers’ choice of ethnic classification method affects substantive findings in adolescent mental health. For example, it is not uncommon to see a statement like “Māori students [6.5%] were more likely than European students [2.7%]…to have attempted suicide in the past 12 months (*OR* = 1.88, 95% CI [1.35–2.60])” (Clark et al. 2018, p. 5). However, many studies do not provide clear information on how ethnicity has been operationalised, so it remains relatively unknown how different ethnic classification methods, such as the ones described in the subsequent section, change the reported “absolute” prevalence rate for an ethnic group (e.g., 6.5% for Māori), and the reported magnitude of relative difference between ethnic groups (e.g., odds ratio [*OR*] of 1.88 between Māori and European). Substantive variation in reported prevalence rates and magnitude of difference can impact conclusions on the level of mental health challenges experienced by each ethnic group, the extent of disparities between ethnic groups, and ultimately, how ethnic inequities are addressed via intervention and policy.

### Classifying Multiple Ethnic Identifications

Ethnic classification methods for multiple ethnic identifications can be divided into two broad categories: *mutually exclusive methods*, where multi-ethnic participants are allocated to a single ethnic group; and *non-mutually exclusive methods*, where multi-ethnic participants are allocated to two or more overlapping ethnic groups. Mutually exclusive methods, particularly ones that prioritise multiple ethnic identifications into broad ethnic groupings, tend to be more popular among applied researchers predominantly because they are easier to incorporate into statistical analysis (Yao et al., 2021). However, there are concerns associated with prioritisation methods because they suppress participants’ multi-ethnic affiliation. Conversely, non-mutually exclusive methods preserve participants’ multi-ethnic affiliation, but are more difficult to implement in statistical analysis.

As the specificities of ethnic classification methods are influenced by a country’s ethno-cultural and socio-political context (Morning, 2008), these methods will be discussed with reference to the broad ethnic groupings in the current study’s context of Aotearoa New Zealand: European (e.g., New Zealand European [New Zealanders with European descent], White American, White British); Māori (Indigenous Peoples); Pacific Peoples (e.g., Sāmoan, Cook Islands Māori, Tongan); Asian (e.g., Chinese, Indian, Filipino); Middle Eastern, Latin American, and African (MELAA); and Other (residual category; Statistics New Zealand, 2005). Note that, except for Māori, these groupings are used for statistical output only, and contain considerable intragroup heterogeneity. The term “multi-ethnic” as used in this paper refers to individuals who self-identify with more than one of these broad ethnic groupings, although it should be noted that multiple identifications can also occur within each grouping (e.g., Sāmoan/Tongan). Aotearoa New Zealand is a valuable context for research on ethnic classification given its comparatively high ethnic diversity and rate of multi-ethnic identification, particularly among younger age groups (Statistics New Zealand, 2020). In addition, the country has devoted considerable attention to ethnic classification in official statistics (Statistics New Zealand, 2004), and is obliged through its founding document, *Te Tiriti o Waitangi* (*The Treaty of Waitangi*)^2^ to ensure equity and address disparities between Māori and non-Māori (Cormack & Robson, 2010).

#### Mutually exclusive methods

Mutually exclusive ethnic classification methods include *sole/combination grouping*, *administrative-prioritisation*, and *self-prioritisation* (in the United States, these are respectively referred to as “multiracial combinations”, “deterministic whole assignment”, and “best race”; Office of Management and Budget [OMB], 2000). Each of these methods assign a participant to one ethnic category only. With sole/combination grouping, mono-ethnic participants are assigned to their sole ethnic group (e.g., sole European, sole Māori, sole Pacific, etc.), and multi-ethnic participants are assigned to their specific ethnic combination (e.g., Māori/European, Pacific/European, Māori/Pacific/European, etc.; Statistics New Zealand, 2005). With administrative-prioritisation, multi-ethnic participants are allocated to a single broad ethnic grouping according to a predetermined hierarchy. The standard hierarchy used in Aotearoa New Zealand is: Māori > Pacific > Asian > MELAA > Other > European (e.g., a Māori/European participant would be prioritised as Māori; Department of Statistics, 1993). With self-prioritisation, multi-ethnic participants are asked in a follow-up question to select one “main” ethnic group (Kukutai & Callister, 2009).

International literature tends to recommend sole/combination grouping because it retains participants’ multi-ethnic identifications and allows more nuanced analysis (Charmaraman et al., 2014; Sanchez et al., 2020). Sole/combination grouping is also an officially recommended output method by Statistics New Zealand (2005), alongside total response grouping (see next section). In contrast, administrative-prioritisation and self-prioritisation suppress multi-ethnic identifications—the former in a way that does not account for participants’ strength of self-affiliation, and the latter in presenting a “forced choice dilemma” to participants (Shih & Sanchez, 2005). However, prioritisation methods tend to be more commonly used than sole/combination grouping in applied research in Aotearoa New Zealand. In particular, administrative-prioritisation tends to be routinely used in the education and health sectors (Yao et al., 2021). This is likely due to a combination of factors, including recognition of Māori rights under *Te Tiriti o Waitangi* (Māori are prioritised in the hierarchy), and avoiding having a large number of ethnic categories, some with small subgroup sizes.

Regardless of the specific ethnic classification method, inclusion of mutually exclusive ethnic groups as an independent variable for statistical analyses is relatively straightforward. For example, in a general linear model (GLM; e.g., analysis of variance [ANOVA], linear regression, logistic regression, multilevel modelling), the process usually involves selecting a reference group (typically the dominant ethnic group, e.g., European), dummy-coding the remaining groups, and then simultaneously entering the set of *k* – 1 ethnicity variables into the model (*k* represents the total number of ethnic groups, the reference group is omitted from the model; Cohen et al., 2003). Interpretation of resulting ethnicity coefficients relative to the reference group is reasonably intuitive, and because outcomes between the dominant ethnic group and minority ethnic groups are directly contrasted, effects of power and privilege versus marginalisation and disadvantage can be inferred.^3^ Mutually exclusive ethnic categories are also well-suited for, and simple to include in, statistical techniques such as chi-square tests of independence, multi-group confirmatory factor analysis (MGCFA), and structural equation modelling (SEM).

#### Non-mutually exclusive methods

Alternatively, multiple ethnic identifications can be outputted using non-mutually exclusive methods, where a multi-ethnic participant is counted in each of the broad ethnic groups they identify with. The officially recommended—and commonly used—non-mutually exclusive ethnic classification method in Aotearoa New Zealand is *total response grouping* (Statistics New Zealand, 2005; known as the “all-inclusive method” in the United States; OMB, 2000). Under total response grouping, ethnicity data are structured as a set of six separate overlapping binary indicators which respectively indicates identification with each broad ethnic grouping (European: yes/no; Māori: yes/no; Pacific: yes/no; Asian: yes/no; MELAA: yes/no; Other: yes/no).

Statistical analyses using total response grouping typically involve a series of regression models containing one binary indicator at a time. Traditionally, each total response ethnic group of interest (*x*) is compared against all the participants who did not identify with that ethnic group (non-*x*; e.g., one model for Māori versus non-Māori, one model for Pacific versus non-Pacific, etc.). This method, hereafter referred to as *original total response*, results in reference groups with high intragroup heterogeneity, making it generally unsuitable for examining the effects of disadvantage and discrimination associated with ethnicity. For example, the reference group (e.g., non-Māori) usually includes participants who are also likely to experience marginalisation (e.g., Pacific, Asian, MELAA), and thus can attenuate observed effects. Another limitation is that the reference group is inconsistent across models.

Therefore, some researchers adopt a *modified total response* approach, where the series of regression models use a consistent reference group. The reference group usually comprises the most privileged ethnic group(s) in the context of investigation. Examples of reference groups used in research in Aotearoa New Zealand include sole European (i.e., those who only identify as European; Clark et al., 2018), sole European/Other (i.e., those who do not identify as Māori, Pacific, Asian, or MELAA; this differs from sole European because it includes responses such as “New Zealander”; Baker et al., 2012), and non-Māori/non-Pacific (i.e., those who do not identify as Māori or Pacific; Davis et al., 2006). While this approach addresses the limitations of the original total response method, it introduces new issues. For instance, because participants who are not in the comparison or reference groups are excluded from analyses, sample sizes are inconsistent across models, and not all available data are used.

Another non-mutually exclusive ethnic classification method, primarily described in the U.S. literature, is *fractional assignment* (OMB, 2000). This method allocates weightings to each ethnic group selected by a participant so that they sum to 1, usually by assuming that a participant equally identifies with each selected group (e.g., for a Māori/European participant, 0.5 weighting will be assigned to Māori, and 0.5 weighting will be assigned to European). Fractional assignment was not considered an appropriate method for the Aotearoa New Zealand context because it evokes parallels with the derogatory historic framing of multi-ethnic Māori through “blood quantum” terms (e.g., “half-caste”, “quarter-caste”), and because Māori notions of *whakapapa* (ancestry) regard a person as fully belonging to each of their ancestries regardless of “blood quantum” (Jackson, 2003).

### Effects of Ethnic Classification on Outcomes

The effects that ethnic classification method have on the analysis of outcomes is a relatively understudied area, especially in adolescent mental health. In Aotearoa New Zealand, the limited existing research has typically used adult datasets (<10% multi-ethnic prevalence) to compare the effects of total response grouping, administrative-prioritisation, and sole/combination grouping on physical health outcomes—for example, smoking status (Boven et al., 2020), mortality (Callister & Blakely, 2004), sexual health (Lachowsky et al., 2020), and health indicators such as heart disease and diabetes (Ministry of Health, 2008). A noteworthy exception is Hobbs et al.’s (2019) study of ethnic classification effects on infectious diseases in early childhood (31% multi-ethnic prevalence), which also examined children’s “self-prioritised” ethnicity as reported by their mother. In general, despite differences in sample and outcome measure, these studies indicate that prevalence rates within ethnic groups as delineated by total response grouping, administrative-prioritisation, and self-prioritisation (via mother report) differ by up to 5% before demographic characteristics are adjusted for. A slightly larger difference was occasionally observed between sole ethnic groups and total response ethnic groups, particularly for Māori (6% in Boven et al.’s [2020] study; 10% in Hobbs et al.’s [2019] study). After adjusting for demographic characteristics such as socioeconomic deprivation, ethnic classification method largely did not alter interpretations of whether an ethnic group was significantly different relative to the European referent (*p* < 0.05), but did at times produce marked variations in the magnitude of effect (e.g., *OR*s differed by up to 0.32 in Lachowsky et al.’s [2020] study; relative risk differed by up to 11.80 in Hobbs et al.’s [2019] study). Overall, the differences observed are noteworthy considering these arose solely due to a change in ethnic classification method.

Existing studies that delineate analyses by sole/combination grouping allow additional insight into the outcomes of multi-ethnic participants relative to their mono-ethnic counterparts. These show that, in Aotearoa New Zealand, socioeconomic and physical health outcomes for combination ethnic groups generally lie between their constituent sole ethnic groups (Boven et al., 2020; Hobbs et al., 2019). U.S. research with adolescents and adults (both < 10% multi-racial prevalence) has observed similar patterns in socioeconomic outcomes (Bratter, 2018), self-rated health (Tabb et al., 2019), and educational outcomes (Cheng & Lively, 2009). In contrast, with mental health outcomes, U.S. research shows that multiracial youth generally tend to have similar or poorer outcomes on measures such as depression (Cheng & Lively, 2009; Fisher et al., 2014) and suicidality (Campbell & Eggerling-Boeck, 2006; Udry et al., 2003), when compared to their monoracial component group with lower psychological wellbeing, even after adjusting for socioeconomic background. The dominant explanation cited in the literature—which attributes these results to heightened identity conflict arising from having multiple heritages—is based on deficit discourse stemming from the anti-miscegenation era in the United States (Shih & Sanchez, 2005). Some contemporary scholars have importantly underscored the need to shift from an individualistic focus to addressing the wider social context where monoracial categories is the “norm”, as this norm contributes to stigma, microaggression, and discrimination towards multiracial individuals (Sanchez et al., 2020; Skinner et al., 2020). In Aotearoa New Zealand, the mental health status of multi-ethnic adolescents relative to their constituent ethnic groups is largely unknown.

## The Current Study

Given the importance of ethnicity in adolescent mental health research and the dearth of information about the effects of ethnic classification methods in multi-ethnic contexts, this study uses a large adolescent dataset from Aotearoa New Zealand to investigate how different ethnic classification methods affect the substantive findings of three adolescent mental health outcomes: (1) overall psychosocial difficulties, (2) deliberate self-harm, and (3) suicide attempt. The study was guided by two research questions. First, how does ethnic classification method affect the *absolute level* of adolescent mental health outcomes reported for *each* ethnic group (Research Question 1)? Second, how does ethnic classification method affect the *relative difference* in adolescent mental health outcomes reported *between* ethnic groups (Research Question 2)?

## Methods

### Data Source

Secondary data from the Youth’12 survey were used for this study. Youth’12 is a nationally representative cross-sectional survey of the health and wellbeing of secondary school students in Aotearoa New Zealand (aged 12–18 years; see Clark et al., 2013). Participants were selected using a two-stage clustered sampling design: one-third of secondary schools in the country were randomly selected, and within each of these schools, 20% of students on the school roll were randomly selected and invited to participate. In smaller schools (<150 students), 30 students from each school were randomly selected to protect confidentiality. Sampling weights were used to adjust for unequal likelihood of selection. Of the 12,503 randomly selected students, 8500 (68%) participated in the survey. Among those who did not participate, the most common reasons were absence from school (22%), refusal to take part (20%), and unavailability due to other school activities (11%; Clark et al., 2013). Reason for non-participation was unavailable for 37% of non-participating students. The online survey was administered in schools via computer tablets, and was available in both English and *te reo Māori* (the Māori language). There was also optional audio voice-over. Ethical approval for Youth’12 was obtained from the University of Auckland Human Participants Ethics Committee (reference 2011/206).

Participants with missing ethnicity data (*n* = 36; 0.4%), and/or other missing demographic data listed in the Measures section (*n* = 192; 2.3%), were omitted from the current study, resulting in an analytic sample of 8275 (2.6% excluded in total). The small proportion of cases omitted (<5%) is unlikely to lead to biased results (Graham, 2009). Table 1 shows the overall demographic characteristics of the analytic sample. Thirty-two percent of the sample identified with more than one broad ethnic group. Demographic characteristics delineated by ethnicity, as classified using each ethnic classification method, are available in Supplementary Table S1.

**Table 1:** Sample demographic characteristics (*N* = 8275)

Demographic characteristic	*n*	%
*Sex*		
Male	3752	45
Female	4523	55
*Age* (*years*)		
≤13	1785	22
14	1843	22
15	1718	21
16	1542	19
≥17	1387	17
*Number of broad ethnic groups*		
1	5630	68
2	2212	27
≥3	433	5
*Urbanicity*		
Main urban	6158	74
Minor urban	916	11
Rural	1201	15
*New Zealand Deprivation Index* (*NZDep*)		
1–2 (lowest deprivation)	1681	20
3–4	1572	19
5–6	1561	19
7–8	1507	18
9–10 (highest deprivation)	1954	24

### Measures

#### Mental health outcomes

##### Overall psychosocial difficulties

Overall psychosocial difficulties was measured using total difficulties score in the Strength and Difficulties Questionnaire (SDQ; R. Goodman et al., 1998). Total difficulties score is the sum of the items in the SDQ’s four difficulties subscales (emotional symptoms, peer problems, hyperactivity-inattention, and conduct problems), and has been found to be predictive of clinician-rated mental health diagnoses (A. Goodman & Goodman, 2009). Each subscale consisted of five items, and each item was rated on a 3-point Likert-type scale (0 = *not true*, 1 = *somewhat true*, 2 = *certainly true*). Negatively-worded items were reverse-scored. This produced a total difficulties score ranging from 0 to 40, with higher scores reflecting greater difficulties. The total score was then standardised to have a sample mean of 0 and standard deviation of 1. There were no partially missing responses, as the Youth’12 survey required participants to answer all the SDQ items (or skip the entire SDQ section).

##### Deliberate self-harm

Deliberate self-harm was a binary variable (0 = *no*, 1 = *yes*) derived from the following question: “During the last 12 months, have you deliberately hurt yourself or done anything you knew might have harmed you (but not kill you)?”. Five response options were provided. *Not at all* was coded as 0; and *yes – once*, *yes – two times*, *yes – 3*–*5 times*, and *more than 5 times* were coded as 1.

##### Suicide attempt

Suicide attempt was a binary variable (0 = *no*, 1 = *yes*) derived from the following question: “During the last 12 months, have you tried to kill yourself (attempted suicide)?”. Four response options were provided. *Not at all* and *not in the last 12 months* were coded as 0; and *once or twice* and *three or more times* were coded as 1.

#### Demographic characteristics

##### Ethnicity

The focal independent variable of ethnicity, classified in five different ways, was based on self-report on two questions. The first question of “Which ethnic group do you belong to? (you may choose as many as you need)” had a check-all-that-apply format with 24 response options (e.g., New Zealand European, English, Australian, Māori, Sāmoan, Cook Island Māori, Filipino, Chinese, Indian, Middle Eastern, Latin American, African, etc.). Responses were aggregated into five broad total response ethnic groupings (European, Māori, Pacific, Asian, and Other),^4^ then outputted in four ways: (1) *sole/combination grouping*, with twelve mutually exclusive dummy-coded categories (sole European [reference], sole Māori, sole Pacific, sole Asian, sole Other, Māori/European, Pacific/European, Asian/European, Māori/Pacific, Māori/Pacific/European, two groups not elsewhere included [NEI; e.g., Māori/Asian, Pacific/Asian], and three or more groups NEI [e.g., Māori/Pacific/Asian]); (2) *original total response grouping*, with five non-mutually exclusive binary indicators that compared each total response ethnic group *x* (European, Māori, Pacific, Asian, and Other) to the reference category of non-*x* (non-European, non-Māori, non-Pacific, non-Asian, and non-Other, respectively); (3) *modified total response grouping*, with four non-mutually exclusive binary indicators that compared each applicable total response ethnic group *x* (Māori, Pacific, Asian, and Other) to the identical reference category of sole European (note total response European was not applicable as a comparison group for this method because the reference group was sole European); and (4) *administrative-prioritisation*, with five mutually exclusive dummy-coded categories, where multiple ethnic responses were prioritised according to the following hierarchy: Māori > Pacific > Asian > Other > European (reference). The final classification method of (5) *self-prioritisation*, was based on the single-selection question of “Which is your main ethnic group (the one you identify with most)?”. The same list of 24 response options was used, with an additional “I can’t choose only one ethnic group” option. Responses were aggregated into six mutually exclusively categories (European [reference], Māori, Pacific, Asian, Other, and “can’t choose”), and then dummy-coded.

##### Sex

Self-reported sex was coded as male (reference) and female.

##### Age

Self-reported age in years was coded as ≤13 (reference), 14, 15, 16, and ≥17.

##### Urbanicity

Urbanicity was derived from participants’ residential meshblock,^5^ and coded as main urban (large urban areas with population ≥ 30,000 [reference]), non-main urban (small-medium urban areas with population between 1000 and 29,999), and rural (population < 1000).

##### Socioeconomic deprivation

Socioeconomic deprivation was measured using the New Zealand Deprivation Index (NZDep; Atkinson et al., 2014), a meshblock-based index constructed using 2013 census data such as income, employment, educational qualification, and home ownership. NZDep was coded into quintiles, from the least deprived 20% of meshblocks (NZDep 1–2 [reference]) to the most deprived 20% of meshblocks (NZDep 9–10).

### Data Analysis

Data were analysed using R version 4.0.2 (R Core Team, 2020). The relationship that the independent variables had with mental health outcomes, with a focus on ethnicity as classified in five different ways, was examined using multiple linear regression for total difficulties score, and binary logistic regression for self-harm and suicide attempt. Twelve separate regression models were specified for each outcome, with each model differing in the way ethnicity was classified (because membership in total response ethnic groups is not mutually exclusive, separate models were specified for each of the five original total response indicators and each of the four modified total response indicators, resulting in nine models; in addition, one model was specified for each of the mutually exclusive ethnic classification methods: sole/combination grouping, administrative-prioritisation, and self-prioritisation). For each regression model, complete case analysis of the dependent variable was used, resulting in slightly different analytic sample sizes (*N* = 7990 [97%] for total difficulties score, 8170 [99%] for self-harm, and 8119 [98%] for suicide attempt). The small proportion of missingness (<5%) meant that complete case analysis was unlikely to lead to biased results (Graham, 2009). Note the modified total response models had smaller analytic sample sizes, as this classification method omits participants who are not in the comparison or reference groups.

From the resulting regression models, the “effects” package in R (Fox & Weisberg, 2019) was used to calculate adjusted mean estimates for total difficulties score, and adjusted prevalence estimates for self-harm and suicide attempt, for each ethnic group as classified by each ethnic classification method. The other independent variables were held constant at the sample average for these calculations. Note the residual ethnic category of “Other” was included in the regressions, but omitted from the figures in the results section, because it is a highly heterogenous group, making it difficult to draw inferences from.

## Results

### Descriptive Statistics

The overall sample mean for total difficulties score was a raw score of 11.37, 95% confidence interval (CI) [11.24, 11.49]. This was converted to a standardised score (*M* = 0, *SD* = 1) so that differences in scores are represented in standard deviation units (equivalent to *z*-scores and Cohen’s [1988] *d*), and hence easier to interpret. The overall sample prevalence for deliberate self-harm in the past 12 months was 23.9, 95% CI [23.0, 24.8%]; and the overall sample prevalence for suicide attempt in the past 12 months was 4.5, 95% CI [4.0, 5.0%]. Table 2 presents the regression estimates for each outcome by sole/combination ethnicity while keeping sex, age, urbanicity, and NZDep constant at the sample average (unadjusted estimates can be found in the rightmost columns of Supplementary Table S1). Note some combination groups (e.g., Māori/Pacific) had relatively small sample sizes, resulting in wider CIs. The significance levels in Table 2 show that, when compared to sole European, mental health outcomes tended to be significantly poorer for sole Māori, as well as for each ethnic combination examined.

**Table 2:** Adjusted^a^ mental health outcomes by sole/combination ethnicity

Ethnic group(s)		Total difficulties score^b^		Self-harm (%)		Suicide attempt (%)	
	*n*	Estimate	95% CI	Estimate	95% CI	Estimate	95% CI
European	3907	−0.07^c^	[−0.11, −0.04]	22.0^c^	[20.7, 23.4]	2.4^c^	[2.0, 3.0]
Māori	288	0.07^*^	[−0.04, 0.19]	22.7^*^	[18.1, 28.0]	4.4^*^	[2.6, 7.2]
Pacific	538	−0.03	[−0.12, 0.06]	21.9	[18.5, 25.8]	5.7^***^	[4.0, 7.9]
Asian	738	−0.08	[−0.16, −0.01]	17.0	[14.4, 19.9]	2.7	[1.7, 4.1]
Other	159	0.03	[−0.12, 0.19]	19.5	[14.0, 26.5]	3.6	[1.6, 7.9]
Māori/European	967	0.08^***^	[0.02, 0.15]	26.7^***^	[24.0, 29.6]	4.4^**^	[3.3, 5.8]
Pacific/European	384	0.07^*^	[−0.03, 0.17]	24.6^*^	[20.5, 29.3]	6.2^***^	[4.2, 8.9]
Asian/European	224	0.06	[−0.07, 0.19]	23.3	[18.1, 29.5]	5.5^**^	[3.1, 9.5]
Māori/Pacific	78	0.25^**^	[0.03, 0.47]	23.7^**^	[15.7, 34.2]	7.6^**^	[3.7, 14.8]
Māori/Pacific/European	125	0.27^***^	[0.09, 0.45]	35.1^***^	[27.0, 44.2]	7.8^***^	[4.3, 13.7]
2 groups NEI	559	0.06^**^	[−0.02, 0.15]	27.8^**^	[24.2, 31.7]	5.7^***^	[4.1, 7.9]
≥3 groups NEI	309	0.33^***^	[0.21, 0.44]	34.8^***^	[29.6, 40.5]	7.9^***^	[5.4, 11.4]

*Note*. Significance levels denote statistically significant difference from the reference group based on multiple linear regression for total difficulties score, and binary logistic regression for self-harm and suicide attempt. *CI* confidence interval, *NEI* not elsewhere included

^a^Adjusted for sex, age, urbanicity, and NZDep. Estimates calculated at the weighted averages of these variables

^b^Total difficulties score was standardised (*M* = 0, *SD* = 1)

^c^Reference group. *p < 0.05. **p < 0.01. ***p < 0.001

At a descriptive level, combination ethnic groups (except Māori/European) tended to have higher total difficulties score, self-harm, and suicide attempt prevalence than their constituent sole ethnic groups. However, as indicated by the degree of overlap between their 95% CIs (Cumming & Finch, 2005), most of these differences were not statistically significant. In contrast, combination ethnic groups’ demographic characteristics (e.g., NZDep) tended to lie between their constituent sole ethnic groups (see Supplementary Table S1).

### Ethnic Classification Effects on Adjusted Estimates

**Fig. 1:**
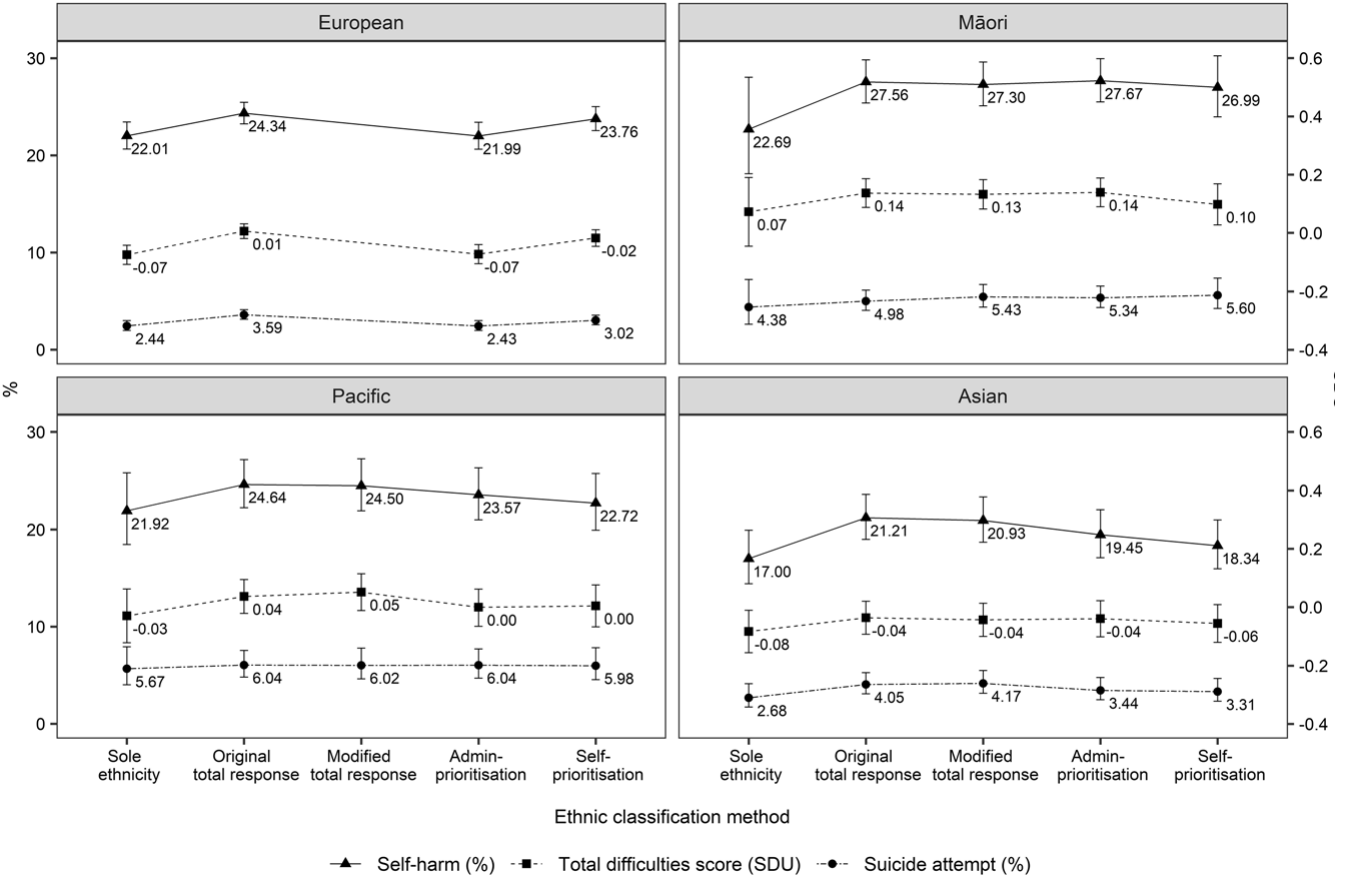
*Adjusted*^*a*^ *Mental Health Outcomes Within Ethnic Groups by Ethnic Classification Method. Note*. Error bars show 95% CIs. Total difficulties score was standardised (*M* = 0, *SD* = 1), so is represented in standard deviation units (SDU). ^a^Adjusted for sex, age, urbanicity, and NZDep. Estimates calculated at the weighted averages of these variables

Figure 1 shows how adjusted estimates for mental health outcomes within each broad ethnic grouping fluctuated by ethnic classification method, as calculated using regression analyses. Due to space, for the sole/combination grouping method, only sole ethnic groups are shown for comparison (outcomes for combination groups can be found in Table 2). In general, sole ethnic groups had the lowest adjusted estimates (except for administratively-prioritised European, which was equivalent to sole European because European is in last position on the prioritisation hierarchy). Conversely, the two total response methods (original and modified) tended to have the highest adjusted estimates,^6^ whereas the two prioritisation methods (administrative and self) tended to have estimates that were slightly lower than the total response methods. The estimates for administratively-prioritised Māori were an exception—these were similar to total response Māori due to its first position on the prioritisation hierarchy. Figure 1 also shows 95% CIs for each point estimate to indicate the range where there is relative certainty the true population value will lie. Note these CIs should not be used to infer statistical difference between ethnic classification methods within an ethnic group, as they do not represent independent groups (i.e., the data are dependent; Cumming & Finch, 2005).

Effect sizes were used to quantify the differences in adjusted estimates within each ethnic group. Cohen’s (1988) *d*, which indicates the standardised difference between two means, was used for total difficulties score. For prevalence differences in deliberate self-harm and suicide attempt, Cohen’s *h* (an effect size for proportions analogous to Cohen’s *d* for means) was used. These effect size measures are appropriate for both independent and dependent groups (Cohen, 1988). For reference, Cohen’s rule of thumb for interpreting *d* and *h* are: 0.20 = small effect, 0.50 = medium effect, and 0.80 = large effect. It is important to note that this interpretation is usually applied to differences between groups (e.g., Māori vs. European), rather than differences due to methodological changes alone (e.g., sole Māori vs. total response Māori), so the benchmarks are overly conservative for the comparisons made here.

In this study, the largest within-group differences resulting from a change in ethnic classification method tended to have an effect size between 0.05 and 0.10. For example, adjusted suicide attempt prevalence for sole European was 2.4%, compared to 3.6% for total response European. This translates to a percentage increase of 50% and had an effect size of *h* = 0.07. For Māori, the largest difference in adjusted suicide attempt prevalence was between sole Māori (4.4%) and self-prioritised Māori (5.6%), equating to a 27% increase and an effect size of *h* = 0.06. The largest within-group difference in effect sizes was observed in Māori self-harm rates—the rate was 22.7% for sole Māori (note relatively large CI) and 27.7% for administratively-prioritised Māori (relatively smaller CI), reflecting a 22% increase and an effect size of *h* = 0.12.

### Ethnic Classification Effects on Subgroup Differences

**Fig. 2:**
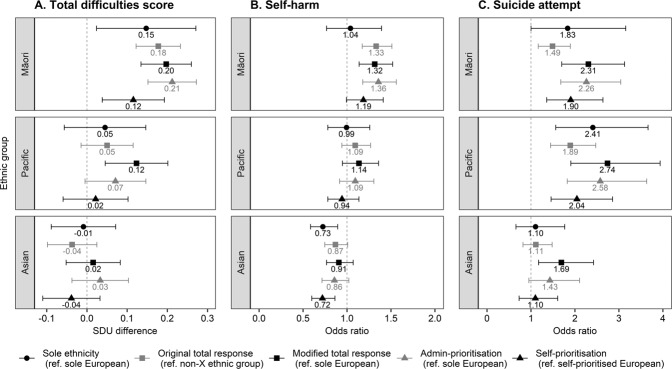
*Partial Regression Coefficients*^*a*^ *for Ethnicity by Ethnic Classification Method and Mental Health Outcome. Note*. Error bars show 95% CIs (note different *x*-axis scale for each outcome). Vertical dotted line indicates the null effect line. European panel is not applicable as it was the reference group in most cases. Total difficulties score was standardised (*M* = 0, *SD* = 1), so group differences are represented in standard deviation units (SDU). ^a^Controlling for sex, age, urbanicity, and NZDep

Next, the effects of ethnic classification method on between-group differences in mental health outcomes, as estimated using regression analyses, were investigated (again, for sole/combination ethnicity, only sole ethnic groups were used for comparison). Figure 2 shows the partial regression coefficients and 95% CIs for ethnicity, after adjusting for sex, age, urbanicity, and NZDep (full results, which include parameter estimates for all demographic characteristics, can be found in Supplementary Tables S2–S7). First, the investigation examined whether a change in ethnic classification method altered the interpretation of whether an ethnic group’s mental health outcome was significantly different to its reference group (*p* < 0.05, indicated by a 95% CI that does *not* cross the dotted null effect line; as above, CIs should not be compared across ethnic classification methods due to their dependency; Cumming & Finch, 2005). The reference group for each classification method is shown on the legend.

The CIs in Fig. 2 show that ethnic classification method did not alter significance interpretations when ethnicity effects were relatively large. For example, suicide attempt prevalence for Māori and Pacific was significantly higher than their respective reference group regardless of the ethnic classification method used (none of these CIs crossed the null effect line). However, when ethnicity effects were smaller, significance interpretations were sometimes inconsistent between ethnic classification methods (some CIs crossed the null effect line, others did not). For example, total difficulties score for Pacific was significantly different from its referent when modified total response was used, but no significant differences were observed under the remaining four ethnic classification methods. Similarly, self-harm for Māori was significantly different from its referent according to total response (both original and modified) and administrative-prioritisation, but not significantly different according to sole ethnicity and self-prioritisation. Note sole ethnicity typically had wider CIs than the other classification methods due to smaller subgroup sizes.

Second, the extent that the magnitude of effects for ethnicity differed by ethnic classification method was examined (indicated by the distance between points within each sub-panel in Fig. 2). For all three mental health outcomes, modified total response and administrative-prioritisation tended to produce the largest ethnicity effects for each ethnic group, whereas sole ethnicity and self-prioritisation tended to produce smaller effects. However, the impact of original total response depended on the outcome—it produced relatively larger effects for self-harm, and relatively smaller effects for total difficulties score and suicide attempt. Differences in the magnitude of effect by ethnic classification method were most substantial for suicide attempt. For example, for both Māori and Pacific, the smallest adjusted *OR* (original total response; 1.49 and 1.89, respectively) and largest *OR* (modified total response; 2.31 and 2.74, respectively) differed by more than 0.80, equivalent to a Cohen’s (1988) *d* effect size of up to 0.25.^7^ For total difficulties score and self-harm, the largest difference in magnitude within each ethnic group was less substantial but still noteworthy (*d* ranged from 0.07 to 0.10, and 0.10 to 0.15, respectively).

The effect of ethnic classification method on the results of the other demographic characteristics was not a key focus of this study. However, a brief examination of the significance patterns at *p* < 0.05 between the different models for each outcome (see Supplementary Tables S2–S7) showed that females and those living in higher NZDep areas (i.e., poorer neighbourhoods) had significantly worse mental health outcomes regardless of the ethnic classification method used. Within each outcome, significant differences by age tended to be relatively consistent across ethnic classification methods. Outcomes were generally not significantly associated with urbanicity regardless of the ethnic classification method used. The only exception was for self-harm, where the modified total response Māori model and the self-prioritised model indicated that those living in rural areas reported significantly lower self-harm rates than those living in urban areas.

## Discussion

Ethnicity is an important variable for adolescent mental health research, but little is known about how different ways of classifying self-identified ethnicity impact the conclusions drawn. The current study investigated how common ethnic classification methods affect the substantive findings of adolescent mental health outcomes in a nationally representative sample in Aotearoa New Zealand with 32% multi-ethnic prevalence. Overall, the results indicate that the majority of adolescents did not have significant mental health concerns. However, around one in five reported deliberate self-harm, and one in twenty reported attempting suicide, in the year prior to the survey. Consistent with existing patterns in Aotearoa New Zealand, Māori and Pacific youth generally tended to have disproportionately inequitable mental health outcomes in comparison to European and Asian youth (Fleming et al., 2020a). However, the current study revealed important nuances by ethnic classification method and outcome measure, demonstrating the influence of methodological decisions on research conclusions in multi-ethnic contexts.

### Ethnic Classification Effects Within Ethnic Groups

Within each broad ethnic grouping, mental health outcomes tended to fluctuate by the ethnic classification method used. The largest differences typically ranged between a Cohen’s (1988) *d* and *h* effect size of 0.05 and 0.10 (the largest observed effect size was 0.12), which is substantial given this was solely due to a change in ethnic classification method within the same sample. Within each ethnic group, sole ethnicity generally produced the most positive mental health outcomes out of the five ethnic classification methods examined (e.g., lower self-harm prevalence), and total response methods (i.e., original and modified total response) generally produced the least positive outcomes (e.g., higher self-harm prevalence). Outcomes as analysed by the prioritisation methods (i.e., administrative-prioritisation and self-prioritisation) tended to lie in-between (note exceptions for administratively-prioritised Māori and European due to their first and last position on the prioritisation hierarchy, respectively). In other words, studies that use different ethnic classification methods can potentially report substantively different descriptive results. For example, using the same dataset, a study which examined adjusted self-harm rates for Māori using sole ethnicity would have reported a rate of 22.7%, whereas a study which used original total response would have reported a rate of 27.6%. Therefore, consistent with previous studies in Aotearoa New Zealand which investigated physical health outcomes among children (Hobbs et al., 2019) and adults (Boven et al., 2020; Ministry of Health, 2008), descriptive statistics of mental health outcomes were influenced by ethnic classification method. However, it is important to note that the exact effect of ethnic classification method depends on the subsample and outcome measure, regardless of whether the outcomes are within a particular domain (e.g., the current study showed that the magnitude of effect differed somewhat across ethnic groups and mental health outcomes), or across different domains (e.g., the current study suggests sole Māori had lower rates of self-harm than total response Māori, whereas Boven et al.’s [2020] study with adults showed that sole Māori had higher rates of tobacco smoking than total response Māori).

Nuance provided through sole/combination grouping suggests that, in this study, the pattern of results by ethnic classification method may be attributable to how multi-ethnic participants were classified. Specifically, total response grouping includes all participants who identified with the specified ethnic group (whether alone [“mono-ethnic”] or in combination [“multi-ethnic”]), the prioritisation methods may only classify some multi-ethnic participants to the ethnic group (e.g., under administrative-prioritisation, the Pacific grouping would exclude Māori/Pacific participants, but include Pacific/European participants), and sole/combination grouping separates sole-ethnic and multi-ethnic participants into specific categories. The proportion of multi-ethnic participants included may lead to different reported outcomes by ethnic classification method because, while differences between multi-ethnic combinations and their constituent ethnic groups were typically not statistically significant, combination groups (with the exception of Māori/European) tended to have poorer mental health outcomes than their constituent groups at a descriptive level. These mental health disparities are similar to patterns observed among adolescents in the United States in regards to depression (Cheng & Lively, 2009; Fisher et al., 2014) and suicidality (Campbell & Eggerling-Boeck, 2006; Udry et al., 2003), and are likely driven by societal stigma (e.g., negative stereotypes) and discrimination (e.g., identity denial or questioning) towards multi-ethnic individuals (Sanchez et al., 2020; Skinner et al., 2020). As above, it is important to note that these patterns may differ across outcomes. For example, consistent with previous research with adults in Aotearoa New Zealand (Boven et al., 2020; Lachowsky et al., 2020) and the United States (Bratter, 2018; Udry et al., 2003), the current study found that, unlike mental health outcomes, the demographic characteristics (e.g., socioeconomic profile) of ethnic combination groups tended to lie between their component ethnic groups.

### Ethnic Classification Effects Between Ethnic Groups

In addition to reporting descriptive statistics by ethnic group, ethnicity data are also frequently used to monitor and address inequities between groups (Fleming et al., 2020a; Mays et al., 2003). When investigating ethnic inequities, interpretations are typically drawn by examining: (1) whether there is a significant difference in outcomes between an ethnic group and a referent (typically the dominant ethnic group), and (2) the magnitude of difference between the groups. Similar to Lachowsky et al.’s (2020) study on the effects of ethnic classification on sexual health outcomes for homosexual men in Aotearoa New Zealand, the current results on adolescent mental health show that conclusions on whether ethnic groups are significantly different can depend on the ethnic classification method and outcome measure. For example, in comparison to the reference group (i.e., non-*x* ethnic group for original total response, self-prioritised European for self-prioritisation, and sole European for all other methods), three of the five classification methods examined showed that Māori youth had significantly higher self-harm prevalence, and one of the five methods showed Pacific youth had significantly higher total difficulties score. In contrast, all five ethnic classification methods examined showed that Māori and Pacific adolescents had higher rates of suicide attempt and that Māori had higher total difficulties score. In addition, the prevalence of self-harm in Pacific youth was not significantly different from their peers regardless of classification method. Based on patterns of ethnic group differences for each outcome, it appears that ethnic classification method is more likely to influence significance interpretations when the magnitude of ethnic differences is small, but interpretations are relatively robust when the magnitude is large. It is also important to note that explanatory power can differ by ethnic classification method. For example, because Māori and Pacific adolescents tend to have higher rates of multi-ethnic identification, there were fewer participants in the respective sole ethnic groups, resulting in larger error margins and less statistical power to detect an effect if it exists (i.e., higher chance of Type II error).

In terms of magnitude of ethnic differences in adolescent mental health, modified total response and administrative-prioritisation tended to result in the largest effects, likely because these methods compare groups that include multi-ethnic participants (e.g., total response Māori, total response Pacific)—who at a descriptive level tended to have poorer mental health outcomes relative to their constituent ethnic groups—to the reference group of sole European. For example, for ethnic differences in suicide attempt for Māori and Pacific youth, changing the reference group from sole European (i.e., modified total response) to non-Māori and non-Pacific (i.e., original total response), respectively, reduced the magnitude of between-group differences by an effect size (*d*) of up to 0.25. This indicates the influence of researchers’ choice of reference group, and has important implications particularly for health and education research in Aotearoa New Zealand, where administrative-prioritisation is routinely used (Yao et al., 2021). Although the referent of sole European may be appropriate for studies interested in the impact of ethnic marginalisation on outcomes (Lachowsky et al., 2020), depending on the outcome, it is possible that observed differences may be partially attributable to contextual factors that are detrimental to multi-ethnic youth, such as multi-ethnic stigma and discrimination (Sanchez et al., 2020; Skinner et al., 2020). These factors can act as confounds in analyses if not explicitly modelled.

Ethnic differences in mental health outcomes tended to be smaller when self-prioritised ethnicity was used. This may partly be due to the inclusion of multi-ethnic participants in both the comparison and reference groups, but because self-prioritisation can arguably be a crude proxy of strength of ethnic affiliation (Kukutai & Callister, 2009), it is also possible that it reflects the protective effect of strong ethnic identity on mental health (Anderson & Mayes, 2010). However, research suggests that self-prioritisation is influenced by contextual factors such as societal stereotypes (Yao et al., 2022). Therefore, if researchers are interested in the effects of ethnic identity, it would be more appropriate to measure this directly, for example, using the Multigroup Ethnic Identity Measure (MEIM; Phinney & Ong, 2007), for each of the ethnic groups identified by a participant.

In summary, researchers’ conclusions about ethnic group differences, both in terms of statistical significance and magnitude of effect, can differ depending on the ethnic classification method. Moreover, the effect of method can differ by outcome measure, even if these measures are all within the mental health domain. Finally, it is important to note that while ethnic classification methods had an influence on the interpretation of ethnic group differences, other demographic characteristics (e.g., sex, age, socioeconomic deprivation) were relatively robust to changes in ethnic classification method.

### Limitations and Future Research

Some limitations of this study need to be noted. First, the study was conducted with a cross-sectional adolescent sample from 2012, as this survey included a question on self-prioritised ethnicity. Although it was a nationally representative sample at the time, the 2019 iteration of the survey shows that the proportion of participants who identified with an Asian ethnic group increased by over 10% from 2012 (Fleming et al., 2020b), and that, consistent with international trends, there has been an overall rise in internalising mental health symptoms (Fleming et al., 2020a). This may have some impact on the generalisability of the study, although initial reports indicate that the rate of multi-ethnic identification, the proportions of total response ethnic groups other than Asian, as well as the relative mental health status of total response ethnic groups (including Asian), were similar across the two cohorts (Fleming et al., 2020a; Fleming et al., 2020b).

Second, total difficulties from the SDQ was examined as a composite score rather than a latent factor with inherent measurement error. While this is a common way of using the well-validated SDQ (Achenbach et al., 2012), the measure does not appear to have been validated among adolescents in Aotearoa New Zealand. Future research could explore ethnic invariance of the SDQ in this age group and context, including whether conclusions differ by ethnic classification method, as this will provide valuable information for researchers who wish to work with ethnicity data under a latent framework.

Third, the focus of the present study was to investigate the effects that ethnic classification method has on the substantive findings of ethnic differences in adolescent mental health outcomes, rather than to explore possible mechanisms underlying these differences. This risks the erroneous interpretation that observed disparities are due to inherent differences rather than factors such as systemic disadvantage experienced by Indigenous and ethnic minority groups. While it was beyond the scope of this study, it is important for research to directly examine causal factors (e.g., ethnic identity and racial discrimination; Crengle et al., 2012) associated with mental health outcomes for specific ethnic groups, in order to inform knowledge, intervention, and policy. It would be interesting for future research to examine whether these results differ by ethnic classification method.

### Implications

Despite these limitations, the effects that ethnic classification method had on substantive outcomes in the current study have important implications for research and practice both in Aotearoa New Zealand and internationally, especially given the rapid growth of the multi-ethnic population worldwide (Aspinall, 2018). This study highlights the influential role that researchers, through their choice of ethnic classification method, have on knowledge construction in the increasingly diverse global context. There are three key research implications from this study. First, it is imperative that researchers critically select their ethnic classification method when undertaking research that includes ethnicity as a variable, and collect ethnicity data in a way that enables the method to be used. Second, researchers need to explicitly state the ethnic classification method chosen, both for transparency and to facilitate the study’s replicability. Ideally, this should be accompanied by the rationale and possible implications the method may have on results. Third, it is important to educate research audiences—ranging from students and researchers, to policymakers and the media—on the complexities of ethnic classification, encourage them to engage more critically with research, and understand that the conclusions drawn can depend on the methods used.

In terms of critically selecting an ethnic classification method, there are two major aspects researchers need to carefully consider. First, researchers should select the method most suitable to the research question and context. It is helpful here to think about who the comparison and reference groups should include. For example, in the Aotearoa New Zealand context, if the research purpose is to examine equity between Māori and European as guaranteed by *Te Tiriti o Waitangi* (*The Treaty of Waitangi*), administrative-prioritisation or modified total response would be more appropriate, because these methods compare everyone who identifies as Māori to those who solely identify as European. In contrast, if the purpose is to examine equity for Pacific Peoples, modified total response would be more appropriate than administrative-prioritisation, because administrative-prioritisation subsumes those who identify as both Pacific and Māori into the Māori category. If the purpose is to examine ethnicity with more nuance, sole/combination grouping may be appropriate, with the caveat that these results need to be interpreted with extreme care. In particular, sole/combination ethnicity tends to be highly fluid, and some categories will have small subgroup sizes, resulting in unequal explanatory power (Callister et al., 2007). Moreover, the relatively individualised results need to be interpreted in a way that does not place blame on individuals or promote deficit thinking, but rather, considers the wider sociohistorical context (e.g., stigma and discrimination towards certain groups).

Second, researchers conducting studies which include Indigenous Peoples should adhere to the four *CARE Principles for Indigenous Data Governance* (Research Data Alliance International Indigenous Data Sovereignty Interest Group, 2019) when selecting their ethnic classification method, such that they: (1) contribute to the *Collective Benefit* of Indigenous Peoples; (2) allow Indigenous Peoples the *Authority to Control* decisions made regarding Indigenous data, including how ethnicity is classified; (3) fulfil their *Responsibility* in sharing how their research supports Indigenous Peoples; and (4) uphold *Ethics* so that research findings using the selected ethnic classification method both minimises harm, and maximises benefit, to Indigenous Peoples. The general principle of minimising harm and maximising benefit should likewise extend to other ethnic groups traditionally marginalised in research (e.g., Pacific and Asian).

While it was not the main focus of this study, the study also has important implications for practice and policy both in Aotearoa New Zealand and other ethnically diverse countries. In particular, the current study generally supports existing research that Māori and Pacific adolescents, and adolescents who identify with more than one ethnic group, tend to have higher mental health needs in comparison to sole European youth (Cheng & Lively, 2009; Fleming et al., 2020a). The literature indicates these inequities are largely due to interpersonal and systemic racism (Williams, 2018). Therefore, at the individual and community levels, it is important that parents, practitioners, schools, and communities support Indigenous, ethnic minority, and multi-ethnic youth during the crucial adolescent developmental phase to develop positive ethnic identity, and equip them with tools to buffer the negative impacts of racial stigma, stereotypes, and discrimination (Benner et al., 2018; Sanchez et al., 2020). Equally, continual attention is needed at the societal level to address the systemic disadvantage and marginalisation that contribute to ethnic inequities in mental health.

## Conclusion

Ethnicity data are critical for monitoring and addressing ethnic inequities, but little is known about how different ethnic classification methods impact substantive findings, particularly in adolescent mental health research. Using a nationally representative adolescent sample with over 30% multi-ethnic prevalence, the current study empirically demonstrates via three mental health outcomes that different ethnic classification methods can lead to different substantive results. Most notably, solely due to the ethnic classification method used, reported mental health outcomes within the same nominal ethnic group varied by an effect size (*d*) of up to 0.12, and the reported magnitude of difference between nominal ethnic groups varied by an effect size (*d*) of up to 0.25. Therefore, it is paramount that researchers exercise criticality and transparency when working with ethnicity data, because their decisions impact the conclusions drawn; which in turn influence intervention, policy, and practice; and ultimately, the health and wellbeing of young people.

## Supplementary information

Supplementary Tables

## Footnotes

1 The social construct of ethnicity is typically termed “race” in the United States, and will be referred to as such when U.S. research is referenced.

2 *Te Tiriti o Waitangi* is a constitutional document between Indigenous Māori and the British Crown, whereby Māori are guaranteed the rights to partnership, participation, and protection.

3 Note some scholars argue that dummy-coding is problematic because the selected reference group is positioned as “normative”, and have advocated for alternative coding methods (e.g., effect coding that compares each ethnic group with the overall sample; Mayhew & Simonoff, 2015).

4 MELAA was included in the “Other” category due to small subgroup size (*n* = 248, 3%).

5 Meshblock is a small geographic unit of approximately 60 to 120 residents based on participants’ address.

6 Although original total response and modified total response included the same group of participants (e.g., total response Māori), their adjusted estimates varied slightly because a different reference group was used (e.g., non-Māori and sole European, respectively).

7 *OR*s were converted to Cohen’s (1988) *d* effect size using the formula of *ln*(*OR*)/1.81 (Chinn, 2000). This allows for comparison of ethnic classification methods on a common scale.

## References

[CR1] Achenbach TM, Rescorla LA, Ivanova MY (2012). International epidemiology of child and adolescent psychopathology I: Diagnoses, dimensions, and conceptual issues. Journal of the American Academy of Child & Adolescent Psychiatry.

[CR2] Adkins DE, Wang V, Dupre ME, van den Oord EJCG, Elder GH (2009). Structure and stress: Trajectories of depressive symptoms across adolescence and young adulthood. Social Forces.

[CR3] Anderson ER, Mayes LC (2010). Race/ethnicity and internalizing disorders in youth: A review. Clinical Psychology Review.

[CR4] Aspinall PJ (2018). What kind of mixed race/ethnicity data is needed for the 2020/21 global population census round: The cases of the UK, USA, and Canada. Ethnic and Racial Studies.

[CR5] Atkinson, J., Salmond, C., & Crampton, P. (2014). *NZDep2013 Index of Deprivation*. University of Otago.

[CR6] Baker MG, Barnard LT, Kvalsvig A, Verrall A, Zhang J, Keall M, Wilson N, Wall T, Howden-Chapman P (2012). Increasing incidence of serious infectious diseases and inequalities in New Zealand: A national epidemiological study. Lancet.

[CR7] Benner AD, Wang Y, Shen Y, Boyle AE, Polk R, Cheng Y-P (2018). Racial/ethnic discrimination and well-being during adolescence: A meta-analytic review. American Psychologist.

[CR8] Boven N, Exeter D, Sporle A, Shackleton N (2020). The implications of different ethnicity categorisation methods for understanding outcomes and developing policy in New Zealand. Kōtuitui: New Zealand Journal of Social Sciences Online.

[CR9] Bratter JL (2018). Multiracial identification and racial gaps: A work in progress. Annals of the American Academy of Political and Social Science.

[CR10] Callister, P., & Blakely, T. (2004). *Ethnic classification, intermarriage, and mortality: Some methodological issues in relation to ethnic comparisons in Aotearoa/New Zealand*. Wellington School of Medicine and Health Sciences.

[CR11] Callister P, Didham R, Potter D, Blakely T (2007). Measuring ethnicity in New Zealand: Developing tools for health outcomes analysis. Ethnicity and Health.

[CR12] Campbell ME, Eggerling-Boeck J (2006). “What about the children?” The psychological and social well-being of multiracial adolescents. The Sociological Quarterly.

[CR13] Charmaraman L, Woo M, Quach A, Erkut S (2014). How have researchers studied multiracial populations? A content and methodological review of 20 years of research. Cultural Diversity and Ethnic Minority Psychology.

[CR14] Cheng S, Lively KJ (2009). Multiracial self-identification and adolescent outcomes: A social psychological approach to the marginal man theory. Social Forces.

[CR15] Chinn S (2000). A simple method for converting an odds ratio to effect size for use in meta-analysis. Statistics in Medicine.

[CR16] Clark, T. C., Fleming, T., Bullen, P., Crengle, S., Denny, S., Dyson, B., Fortune, S., Peiris-John, R., Robinson, E., Rossen, F., Sheridan, J., Teevale, T., Utter, J., & The Adolescent Health Research Group. (2013). *Youth’12 prevalence tables: The health and wellbeing of New Zealand secondary school students in 2012*. The University of Auckland.

[CR17] Clark TC, Le Grice J, Moselen E, Fleming T, Crengle S, Tiatia-Seath J, Lewycka S (2018). Health and wellbeing of Māori secondary school students in New Zealand: Trends between 2001, 2007 and 2012. Australian and New Zealand Journal of Public Health.

[CR18] Cohen, J. (1988). *Statistical power analysis for the behavioral sciences* (2nd ed.). Lawrence Erlbaum Associates.

[CR19] Cohen, J., Cohen, P., West, S. G., & Aiken, L. S. (2003). *Applied multiple regression/correlation analysis for the behavioral sciences* (3rd ed.). Lawrence Erlbaum Associates.

[CR20] Cormack, D., & Robson, C. (2010). *Classification and output of multiple ethnicities: Considerations for Māori health*. Te Rōpū Rangahau Hauora a Eru Pōmare.

[CR21] Crengle S, Robinson E, Ameratunga S, Clark T, Raphael D (2012). Ethnic discrimination prevalence and associations with health outcomes: Data from a nationally representative cross-sectional survey of secondary school students in New Zealand. BMC Public Health.

[CR22] Cumming G, Finch S (2005). Inference by eye: Confidence intervals and how to read pictures of data. American Psychologist.

[CR23] Davis P, Lay-Yee R, Dyall L, Briant R, Sporle A, Brunt D, Scott A (2006). Quality of hospital care for Māori patients in New Zealand: Retrospective cross-sectional assessment. Lancet.

[CR24] Department of Statistics. (1993). *New Zealand standard classification of ethnicity*. Author.

[CR25] Fisher S, Reynolds JL, Hsu W-W, Barnes J, Tyler K (2014). Examining multiracial youth in context: Ethnic identity development and mental health outcomes. Journal of Youth and Adolescence.

[CR27] Fleming, T., Tiatia-Seath, J., Peiris-John, R., Sutcliffe, K., Archer, D., Bavin, L., Crengle, S., & Clark, T. (2020a). *Youth19 Rangatahi Smart Survey, initial findings: Hauora hinengaro/emotional and mental health*. The University of Auckland and Victoria University of Wellington.

[CR26] Fleming, T., Peiris-John, R., Crengle, S., Archer, D., Sutcliffe, K., Lewycka, S., & T. C. (2020b). *Youth19 Rangatahi Smart Survey, initial findings: Introduction and methods*. The University of Auckland and Victoria University of Wellington.

[CR28] Fortune, S., Watson, P., Robinson, E., Fleming, T., Merry, S., & Denny, S. (2010). *Youth’07: The health and wellbeing of secondary school students in New Zealand: Suicide behaviours and mental health in 2001 and 2007*. The University of Auckland.

[CR29] Fox, J., & Weisberg, S. (2019). *An R Companion to Applied Regression* (3rd ed.). Sage.

[CR30] Gillborn D, Warmington P, Demack S (2018). QuantCrit: Education, policy, ‘big data’ and principles for a critical race theory of statistics. Race Ethnicity and Education.

[CR31] Goodman A, Goodman R (2009). Strengths and Difficulties Questionnaire as a dimensional measure of child mental health. Journal of the American Academy of Child & Adolescent Psychiatry.

[CR32] Goodman R, Meltzer H, Bailey V (1998). The Strengths and Difficulties Questionnaire: A pilot study on the validity of the self-report version. European Child & Adolescent Psychiatry.

[CR33] Graham JW (2009). Missing data analysis: Making it work in the real world. Annual Review of Psychology.

[CR34] Hobbs, M. R., Atatoa Carr, P., Fa’Alili-Fidow, J., Pillai, A., Morton, S. M. B., & Grant, C. C. (2019). How differing methods of ascribing ethnicity and socio-economic status affect risk estimates for hospitalisation with infectious disease. *Epidemiology and Infection*, *147*. 10.1017/S0950268818002935. 10.1017/S0950268818002935 PMC6518588 30421688

[CR35] Jackson M (2003). The part-Maori syndrome. Mana.

[CR36] Kessler RC, Avenevoli S, Costello EJ, Georgiades K, Green JG, Gruber MJ, He J, Koretz D, McLaughlin KA, Petukhova M, Sampson NA, Zaslavsky AM, Merikangas KR (2012). Prevalence, persistence, and sociodemographic correlates of DSM-IV disorders in the National Comorbidity Survey Replication Adolescent Supplement. Archives of General Psychiatry.

[CR37] Kukutai T, Callister P (2009). A “main” ethnic group? Ethnic self-prioritisation among New Zealand youth. Social Policy Journal of New Zealand.

[CR38] Lachowsky NJ, Saxton PJW, Dickson NP, Hughes AJ, Jones RG, Clark TC, Ho E, Summerlee AJS, Dewey CE (2020). Ethnicity classification systems for public health surveys: Experiences from HIV behavioural surveillance among men who have sex with men. BMC Public Health.

[CR39] Mayhew MJ, Simonoff JS (2015). Non-white, no more: Effect coding as an alternative to dummy coding with implications for higher education researchers. Journal of College Student Development.

[CR40] Mays VM, Ponce NA, Washington DL, Cochran SD (2003). Classification of race and ethnicity: Implications for public health. Annual Review of Public Health.

[CR41] Ministry of Health. (2008). *Presenting ethnicity: Comparing prioritised and total response ethnicity in descriptive analyses of New Zealand Health Monitor surveys*. Author.

[CR42] Morning A (2008). Ethnic classification in global perspective: A cross-national survey of the 2000 census round. Population Research and Policy Review.

[CR43] Office of Management and Budget. (2000). *Provisional guidance on the implementation of the 1997 standards for the collection of federal data on race and ethnicity*. Executive Office of the President.

[CR44] Phinney JS, Ong AD (2007). Conceptualization and measurement of ethnic identity: Current status and future directions. Journal of Counseling Psychology.

[CR45] R Core Team. (2020). *R: A language and environment for statistical computing*. R Foundation for Statistical Computing. https://www.r-project.org.

[CR46] Research Data Alliance International Indigenous Data Sovereignty Interest Group. (2019). *CARE principles for Indigenous data governance*. The Global Indigenous Data Alliance. https://www.gida-global.org.

[CR47] Sanchez DT, Gaither SE, Albuja AF, Eddy Z (2020). How policies can address multiracial stigma. Policy Insights from the Behavioral and Brain Sciences.

[CR48] Shih M, Sanchez DT (2005). Perspectives and research on the positive and negative implications of having multiple racial identities. Psychological Bulletin.

[CR49] Skinner AL, Perry SP, Gaither S (2020). Not quite monoracial: Biracial stereotypes explored. Personality and Social Psychology Bulletin.

[CR50] Statistics New Zealand. (2004). *Report of the review of the measurement of ethnicity*. Author.

[CR51] Statistics New Zealand. (2005). *Statistical standard for ethnicity*. Author.

[CR52] Statistics New Zealand. (2020). *2018 Census ethnic group summaries*. Author. https://www.stats.govt.nz/tools/2018-census-ethnic-group-summaries.

[CR53] Tabb KM, Gavin AR, Smith DC, Huang H (2019). Self-rated health among multiracial young adults in the United States: Findings from the add health study. Ethnicity & Health.

[CR54] Thapar A, Collishaw S, Pine DS, Thapar AK (2012). Depression in adolescence. Lancet.

[CR55] Udry JR, Li RM, Hendrickson-Smith J (2003). Health and behavior risks of adolescents with mixed-race identity. American Journal of Public Health.

[CR56] Williams DR (2018). Stress and the mental health of populations of color: Advancing our understanding of race-related stressors. Journal of Health and Social Behavior.

[CR57] Yao ES, Meissel K, Bullen P, Atatoa Carr P, Clark TC, Morton SMB (2021). Classifying multiple ethnic identifications: Methodological effects on child, adolescent, and adult ethnic distributions. Demographic Research.

[CR58] Yao ES, Meissel K, Bullen P, Clark TC, Atatoa Carr P, Tiatia-Seath J, Peiris-John R, Morton SMB (2022). Demographic discrepancies between administrative-prioritisation and self-prioritisation of multiple ethnic identifications. Social Science Research.

