# Supplementary material for: Effects of Ethnic Classification on Substantive Findings in Adolescent Mental Health Outcomes
Source: J Youth Adolesc. 2022 Apr 19;51(8):1581–96. doi: 10.1007/s10964-022-01612-6 (PMC9232462; doi:10.1007/s10964-022-01612-6)
Supplement: Supplementary file 1 — Supplementary Tables [file 10964_2022_1612_MOESM1_ESM.pdf]

Supplementary Material

Yao, E. S., Bullen, P., Meissel, K., Tiatia, J., Fleming, T., & Clark, T. C. (2022). Effects of ethnic classification on substantive findings in adolescent mental health outcomes. *Journal of Youth and Adolescence*. https://doi.org/10.1007/s10964-022-01612-6

Supplementary Table S1

Demographic Characteristics and Mental Health Outcomes by Ethnicity

		Demographic characteristic							Mental health outcome[a]		
		Age (years)	Sex		Urbanicity			NZDep decile	Total difficulties score[b]	Self-harm	Suicide attempt
			Male	Female	Main urban	Minor urban	Rural				
Ethnic group(s)	*n*	*M (SD)*	*n* (%)	*n* (%)	*n* (%)	*n* (%)	*n* (%)	*M (SD)*	*M (SD)*	*n* (%)	*n* (%)
Overall	8275	14.89 (1.47)	3752 (45)	4523 (55)	6158 (74)	916 (11)	1201 (15)	5.63 (2.97)	0.00 (1.00)	1953 (24)	364 (4)
**Sole/combination grouping**											
European	3907	14.94 (1.46)	1730 (44)	2177 (56)	2582 (66)	525 (13)	800 (20)	4.47 (2.62)	-0.10 (1.01)	853 (22)	103 (3)
Māori	288	14.65 (1.42)	146 (51)	142 (49)	178 (62)	60 (21)	50 (17)	7.83 (2.37)	0.11 (0.93)	66 (24)	16 (6)
Pacific	538	14.84 (1.46)	224 (42)	314 (58)	517 (96)	S (4)	<10 (0)	8.95 (1.67)	0.06 (0.93)	131 (25)	43 (8)
Asian	738	15.26 (1.53)	367 (50)	371 (50)	700 (95)	19 (3)	18 (2)	5.41 (2.71)	-0.08 (0.85)	128 (17)	21 (3)
Other	159	15.24 (1.64)	74 (47)	85 (53)	142 (90)	S (6)	<10 (4)	6.27 (2.67)	0.05 (0.96)	31 (20)	<10 (4)
Māori/European	967	14.75 (1.42)	457 (47)	510 (53)	625 (65)	163 (17)	179 (18)	6.27 (2.81)	0.10 (0.98)	262 (27)	49 (5)
Pacific/European	384	14.70 (1.50)	179 (47)	205 (53)	350 (91)	16 (4)	17 (4)	7.86 (2.52)	0.12 (1.00)	98 (26)	29 (8)
Asian/European	224	15.00 (1.70)	106 (47)	118 (53)	199 (89)	13 (6)	13 (6)	4.90 (2.63)	0.04 (1.02)	51 (23)	12 (6)
Māori/Pacific	78	14.59 (1.32)	33 (42)	45 (58)	63 (81)	S (14)	<10 (5)	7.95 (2.30)	0.31 (1.04)	21 (26)	<10 (10)
Māori/Pacific/European	125	14.47 (1.36)	57 (46)	68 (54)	95 (76)	13 (11)	17 (14)	6.92 (2.67)	0.30 (1.04)	43 (37)	11 (9)
2 groups NEI	559	14.74 (1.43)	236 (42)	323 (58)	467 (83)	37 (7)	56 (10)	5.97 (2.94)	0.08 (0.99)	161 (29)	38 (7)
≥3 groups NEI	309	14.70 (1.41)	144 (47)	165 (53)	240 (78)	28 (9)	41 (13)	6.80 (2.89)	0.35 (1.09)	108 (36)	28 (10)
**Total response grouping**											
European	6209	14.86 (1.46)	2806 (45)	3403 (55)	4312 (69)	783 (13)	1113 (18)	5.14 (2.86)	-0.01 (1.02)	1500 (24)	246 (4)
Māori	1669	14.70 (1.41)	792 (47)	877 (53)	1107 (66)	274 (16)	288 (17)	6.73 (2.78)	0.16 (1.00)	469 (29)	105 (6)
Pacific	1422	14.75 (1.44)	610 (43)	812 (57)	1299 (91)	71 (5)	52 (4)	8.16 (2.33)	0.12 (0.98)	379 (27)	119 (9)
Asian	1256	15.12 (1.56)	592 (47)	664 (53)	1169 (93)	46 (4)	41 (3)	5.68 (2.79)	-0.03 (0.93)	272 (22)	58 (5)
Other	839	14.77 (1.48)	378 (45)	461 (55)	673 (80)	66 (8)	100 (12)	6.07 (2.93)	0.19 (1.01)	244 (30)	57 (7)
**Administrative-prioritisation**											
European	3907	14.94 (1.46)	1730 (44)	2177 (56)	2582 (66)	525 (13)	800 (20)	4.47 (2.62)	-0.10 (1.01)	853 (22)	103 (3)
Māori	1669	14.70 (1.41)	792 (47)	877 (53)	1107 (66)	274 (16)	288 (17)	6.73 (2.78)	0.16 (1.00)	469 (29)	105 (6)
Pacific	1180	14.80 (1.46)	503 (43)	677 (57)	1110 (94)	45 (4)	25 (2)	8.33 (2.24)	0.07 (0.96)	301 (26)	95 (8)
Asian	1036	15.18 (1.58)	509 (49)	527 (51)	968 (93)	34 (3)	34 (3)	5.35 (2.71)	-0.04 (0.91)	203 (20)	37 (4)
Other	484	14.80 (1.50)	219 (45)	265 (55)	392 (81)	37 (8)	54 (11)	5.31 (2.78)	0.11 (0.98)	128 (27)	25 (5)
**Self-prioritisation**											
European	5181	14.88 (1.46)	2323 (45)	2859 (55)	3508 (68)	676 (13)	998 (19)	4.76 (2.73)	-0.04 (1.02)	1215 (24)	169 (3)
Māori	832	14.65 (1.38)	401 (48)	430 (52)	528 (64)	154 (18)	150 (18)	7.44 (2.47)	0.14 (0.97)	231 (29)	57 (7)
Pacific	967	14.81 (1.44)	400 (41)	568 (59)	929 (96)	S (3)	<10 (1)	8.76 (1.89)	0.11 (0.94)	250 (26)	84 (9)
Asian	949	15.23 (1.57)	474 (50)	475 (50)	898 (95)	26 (3)	25 (3)	5.52 (2.71)	-0.06 (0.89)	177 (19)	33 (4)
Other	239	15.11 (1.63)	117 (49)	122 (51)	212 (89)	15 (6)	12 (5)	6.00 (2.73)	0.03 (0.93)	50 (22)	15 (7)
Can't choose one ethnic group	107	14.60 (1.53)	37 (35)	70 (65)	84 (78)	13 (12)	10 (9)	5.61 (3.08)	0.12 (1.03)	30 (29)	<10 (6)

Note. Cell counts less than 10 are suppressed as "<10". Secondary suppression (S) was applied to the next smallest cell so that the suppressed cell cannot be recalculated. NEI = not elsewhere included.

[a]Complete case analysis used (total difficulties N = 7,990 [97%]; self-harm N = 8,170 [99%]; suicide attempt N = 8,119 [98%]).

[b]Total difficulties score was standardised (M = 0, SD = 1).

Supplementary Table S2

Multiple Linear Regression Coefficients for Total Difficulties Score[a] Using Non-Mutually Exclusive Ethnic Classification Methods

Variable	European model			Māori model			Pacific model			Asian model			Other ethnicities model		
	B	*SE*	*p*	*B*	*SE*	*p*	*B*	*SE*	*p*	*B*	*SE*	*p*	*B*	*SE*	*p*
**Original total response models**															
Intercept	-0.33	0.04	<.001	-0.32	0.04	<.001	-0.30	0.04	<.001	-0.29	0.04	<.001	-0.31	0.04	<.001
Female (ref. male)	0.19	0.02	<.001	0.19	0.02	<.001	0.19	0.02	<.001	0.19	0.02	<.001	0.19	0.02	<.001
Age 14 (ref. age ≤13 years)	0.05	0.03	.152	0.05	0.03	.152	0.05	0.03	.152	0.05	0.03	.152	0.05	0.03	.163
Age 15 (ref. age ≤13 years)	0.12	0.03	.001	0.12	0.03	<.001	0.12	0.03	.001	0.12	0.03	.001	0.12	0.03	<.001
Age 16 (ref. age ≤13 years)	0.13	0.03	<.001	0.13	0.03	<.001	0.13	0.03	<.001	0.13	0.03	<.001	0.13	0.03	<.001
Age ≥17 (ref. age ≤13 years)	0.02	0.04	.501	0.03	0.04	.348	0.02	0.04	.526	0.02	0.04	.506	0.03	0.04	.485
Minor urban (ref. main urban)	0.01	0.04	.814	0.00	0.04	.985	0.03	0.04	.486	0.01	0.04	.746	0.02	0.04	.485
Rural (ref. main urban)	-0.05	0.03	.090	-0.06	0.03	.057	-0.04	0.03	.193	-0.05	0.03	.103	-0.04	0.03	.172
NZDep3–4 (ref. NZDep1–2)	0.07	0.04	.051	0.06	0.03	.073	0.06	0.03	.074	0.07	0.04	.057	0.06	0.03	.081
NZDep5–6 (ref. NZDep1–2)	0.12	0.04	.001	0.10	0.04	.003	0.11	0.04	.002	0.12	0.04	.001	0.11	0.04	.001
NZDep7–8 (ref. NZDep1–2)	0.18	0.04	<.001	0.15	0.04	<.001	0.17	0.04	<.001	0.18	0.04	<.001	0.17	0.04	<.001
NZDep9–10 (ref. NZDep1–2)	0.28	0.04	<.001	0.23	0.03	<.001	0.24	0.04	<.001	0.26	0.03	<.001	0.26	0.03	<.001
European (ref. non-European)	0.05	0.03	.095		-			-			-			-	
Māori (ref. non-Māori)		-		0.18	0.03	<.001		-			-			-	
Pacific (ref. non-Pacific)		-			-		0.05	0.03	.129		-			-	
Asian (ref. non-Asian)		-			-			-		-0.04	0.03	.241		-	
Other (ref. non-Other)		-			-			-			-		0.20	0.04	<.001
**Modified total response models**															
Intercept				-0.38	0.04	<.001	-0.31	0.04	<.001	-0.35	0.04	<.001	-0.37	0.04	<.001
Female (ref. male)				0.22	0.03	<.001	0.16	0.03	<.001	0.19	0.03	<.001	0.20	0.03	<.001
Age 14 (ref. age ≤13 years)				0.05	0.04	.181	0.01	0.04	.784	0.03	0.04	.533	0.05	0.04	.296
Age 15 (ref. age ≤13 years)				0.15	0.04	<.001	0.12	0.04	.007	0.14	0.04	.001	0.16	0.05	.001
Age 16 (ref. age ≤13 years)				0.15	0.04	<.001	0.15	0.04	.001	0.17	0.04	<.001	0.18	0.05	<.001
Age ≥17 (ref. age ≤13 years)				0.00	0.04	.974	-0.02	0.05	.710	0.08	0.04	.066	0.03	0.05	.594
Minor urban (ref. main urban)				-0.01	0.04	.756	0.03	0.05	.573	-0.03	0.05	.539	0.01	0.05	.821
Rural (ref. main urban)		-[b]		-0.06	0.04	.089	-0.05	0.04	.210	-0.08	0.04	.043	-0.05	0.04	.202
NZDep3–4 (ref. NZDep1–2)				0.07	0.04	.107	0.05	0.04	.254	0.05	0.04	.245	0.06	0.04	.127
NZDep5–6 (ref. NZDep1–2)				0.13	0.04	.002	0.10	0.04	.026	0.11	0.04	.005	0.11	0.04	.011
NZDep7–8 (ref. NZDep1–2)				0.19	0.04	<.001	0.17	0.05	<.001	0.13	0.04	.004	0.16	0.05	.001
NZDep9–10 (ref. NZDep1–2)				0.25	0.05	<.001	0.20	0.05	<.001	0.28	0.05	<.001	0.24	0.05	<.001
Māori (ref. sole European)				0.20	0.03	<.001		-			-			-	
Pacific (ref. sole European)					-		0.12	0.04	.002		-			-	
Asian (ref. sole European)					-			-		0.02	0.03	.650		-	
Other (ref. sole European)					-			-			-		0.25	0.04	<.001

[a]Total difficulties score was standardised (*M* = 0, *SD* = 1).

[b]Model not applicable as the reference group for modified total response is sole European.

Supplementary Table S3

Multiple Linear Regression Coefficients for Total Difficulties Score[a] Using Mutually Exclusive Ethnic Classification Methods

Variable	Sole/combination model			Administrative-prioritisation model			Self-prioritisation model		
	B	*SE*	*p*	*B*	*SE*	*p*	*B*	*SE*	*p*
Intercept	-0.34	0.04	<.001	-0.34	0.04	<.001	-0.30	0.04	<.001
Female (ref. male)	0.19	0.02	<.001	0.19	0.02	<.001	0.19	0.02	<.001
Age 14 (ref. age ≤13 years)	0.05	0.03	.165	0.05	0.03	.167	0.05	0.03	.163
Age 15 (ref. age ≤13 years)	0.12	0.03	<.001	0.12	0.03	<.001	0.12	0.03	.001
Age 16 (ref. age ≤13 years)	0.14	0.03	<.001	0.14	0.03	<.001	0.13	0.03	<.001
Age ≥17 (ref. age ≤13 years)	0.04	0.04	.265	0.03	0.04	.336	0.03	0.04	.426
Minor urban (ref. main urban)	0.03	0.04	.472	0.02	0.04	.655	0.01	0.04	.809
Rural (ref. main urban)	-0.04	0.03	.177	-0.05	0.03	.138	-0.06	0.03	.088
NZDep3–4 (ref. NZDep1–2)	0.06	0.03	.115	0.06	0.03	.113	0.06	0.04	.066
NZDep5–6 (ref. NZDep1–2)	0.10	0.04	.007	0.10	0.04	.007	0.11	0.04	.002
NZDep7–8 (ref. NZDep1–2)	0.13	0.04	<.001	0.13	0.04	<.001	0.16	0.04	<.001
NZDep9–10 (ref. NZDep1–2)	0.19	0.04	<.001	0.20	0.04	<.001	0.24	0.04	<.001
Māori (ref. European[b])	0.15	0.06	.019	0.21	0.03	<.001	0.12	0.04	.003
Pacific (ref. European[b])	0.05	0.05	.384	0.07	0.04	.066	0.02	0.04	.601
Asian (ref. European[b])	-0.01	0.04	.834	0.03	0.04	.356	-0.04	0.04	.287
Other (ref. European[b])	0.11	0.08	.189	0.19	0.05	<.001	0.04	0.07	.556
Māori/European (ref. European[b])	0.16	0.04	<.001		-			-	
Pacific/European (ref. European[b])	0.14	0.06	.011		-			-	
Asian/European (ref. European[b])	0.13	0.07	.054		-			-	
Māori/Pacific (ref. European[b])	0.32	0.12	.005		-			-	
Māori/Pacific/European (ref. European[b])	0.35	0.09	<.001		-			-	
2 groups NEI (ref. European[b])	0.14	0.05	.003		-			-	
≥3 groups NEI (ref. European[b])	0.40	0.06	<.001		-			-	
Can't choose one ethnic group (ref. European[b])		-			-		0.12	0.10	.231

Note. NEI = not elsewhere included.

[a]Total difficulties score was standardised (*M* = 0, *SD* = 1).

[b]For the sole/combination and administrative-prioritisation models, the referent is *sole* European; for the self-prioritisation model, the referent is *self-prioritised* European.

Supplementary Table S4

Binary Logistic Regression Coefficients for Self-Harm[a] *Using Non-Mutually Exclusive Ethnic Classification Methods*

	European model			Māori model			Pacific model			Asian model			Other ethnicities model		
Variable	*Exp(B)*	*SE*	*p*	*Exp(B)*	*SE*	*p*	*Exp(B)*	*SE*	*p*	*Exp(B)*	*SE*	*p*	*Exp(B)*	*SE*	*p*
**Original total response models**															
Intercept	0.13	0.11	<.001	0.15	0.09	<.001	0.16	0.09	<.001	0.16	0.09	<.001	0.15	0.09	<.001
Female (ref. male)	1.90	0.05	<.001	1.90	0.05	<.001	1.89	0.05	<.001	1.89	0.05	<.001	1.89	0.05	<.001
Age 14 (ref. age ≤13 years)	1.18	0.08	.043	1.18	0.08	.044	1.18	0.08	.044	1.18	0.08	.044	1.17	0.08	.048
Age 15 (ref. age ≤13 years)	1.28	0.08	.002	1.28	0.08	.002	1.27	0.08	.003	1.27	0.08	.003	1.28	0.08	.003
Age 16 (ref. age ≤13 years)	1.19	0.08	.035	1.20	0.08	.032	1.18	0.08	.045	1.19	0.08	.040	1.19	0.08	.038
Age ≥17 (ref. age ≤13 years)	1.00	0.09	.981	1.00	0.09	.957	0.98	0.09	.861	0.99	0.09	.924	0.99	0.09	.896
Minor urban (ref. main urban)	0.97	0.08	.742	0.99	0.08	.882	1.03	0.08	.699	1.00	0.08	.972	1.03	0.08	.708
Rural (ref. main urban)	0.87	0.08	.080	0.88	0.08	.111	0.91	0.08	.260	0.89	0.08	.128	0.91	0.08	.242
NZDep3–4 (ref. NZDep1–2)	1.18	0.09	.051	1.16	0.09	.090	1.16	0.09	.092	1.17	0.09	.069	1.15	0.09	.098
NZDep5–6 (ref. NZDep1–2)	1.25	0.09	.009	1.20	0.09	.036	1.21	0.09	.027	1.23	0.09	.017	1.22	0.09	.024
NZDep7–8 (ref. NZDep1–2)	1.48	0.09	<.001	1.35	0.09	<.001	1.39	0.09	<.001	1.43	0.09	<.001	1.39	0.09	<.001
NZDep9–10 (ref. NZDep1–2)	1.57	0.08	<.001	1.36	0.08	<.001	1.38	0.09	<.001	1.44	0.08	<.001	1.42	0.08	<.001
European (ref. non-European)	1.27	0.07	<.001		-			-			-			-	
Māori (ref. non-Māori)		-		1.33	0.06	<.001		-			-			-	
Pacific (ref. non-Pacific)		-			-		1.09	0.08	.238		-			-	
Asian (ref. non-Asian)		-			-			-		0.87	0.08	.066		-	
Other (ref. non-Other)		-			-			-			-		1.37	0.08	<.001
**Modified total response models**															
Intercept				0.14	0.11	<.001	0.14	0.11	<.001	0.14	0.11	<.001	0.13	0.12	<.001
Female (ref. male)				1.99	0.07	<.001	1.79	0.07	<.001	1.83	0.07	<.001	1.89	0.07	<.001
Age 14 (ref. age ≤13 years)				1.16	0.10	.131	1.15	0.10	.171	1.11	0.11	.350	1.30	0.11	.016
Age 15 (ref. age ≤13 years)				1.43	0.10	<.001	1.36	0.10	.003	1.32	0.11	.009	1.47	0.11	<.001
Age 16 (ref. age ≤13 years)				1.24	0.10	.034	1.37	0.10	.002	1.25	0.11	.041	1.40	0.11	.003
Age ≥17 (ref. age ≤13 years)				1.03	0.11	.772	1.08	0.11	.512	1.16	0.11	.183	1.18	0.12	.169
Minor urban (ref. main urban)				0.93	0.09	.470	1.00	0.11	.982	0.95	0.11	.623	1.03	0.11	.793
Rural (ref. main urban)		-[b]		0.81	0.09	.017	0.88	0.10	.210	0.88	0.10	.205	0.92	0.09	.391
NZDep3–4 (ref. NZDep1–2)				1.19	0.10	.086	1.22	0.11	.063	1.19	0.10	.085	1.20	0.10	.071
NZDep5–6 (ref. NZDep1–2)				1.30	0.10	.009	1.33	0.11	.008	1.34	0.10	.004	1.24	0.11	.040
NZDep7–8 (ref. NZDep1–2)				1.55	0.10	<.001	1.58	0.11	<.001	1.56	0.11	<.001	1.53	0.11	<.001
NZDep9–10 (ref. NZDep1–2)				1.48	0.11	<.001	1.47	0.12	.001	1.69	0.12	<.001	1.51	0.12	.001
Māori (ref. sole European)				1.32	0.07	<.001		-			-			-	
Pacific (ref. sole European)					-		1.14	0.09	.165		-			-	
Asian (ref. sole European)					-			-		0.91	0.08	.253		-	
Other (ref. sole European)					-			-			-		1.41	0.09	<.001

[a]0 = no self-harm in the past 12 months; 1 = self-harm in the past 12 months.

[b]Model not applicable as the reference group for modified total response is sole European.

Supplementary Table S5

Binary Logistic Regression Coefficients for Self-Harm[a] Using Mutually Exclusive Ethnic Classification Methods

Variable	Sole/combination model			Administrative-prioritisation model			Self-prioritisation model		
	Exp(B)	*SE*	*p*	*Exp(B)*	*SE*	*p*	*Exp(B)*	*SE*	*p*
Intercept	0.15	0.09	<.001	0.15	0.09	<.001	0.16	0.09	<.001
Female (ref. male)	1.89	0.05	<.001	1.90	0.05	<.001	1.89	0.05	<.001
Age 14 (ref. age ≤13 years)	1.17	0.08	.050	1.17	0.08	.049	1.17	0.08	.045
Age 15 (ref. age ≤13 years)	1.30	0.08	.001	1.28	0.08	.002	1.28	0.08	.002
Age 16 (ref. age ≤13 years)	1.22	0.08	.017	1.21	0.08	.023	1.21	0.08	.026
Age ≥17 (ref. age ≤13 years)	1.04	0.09	.669	1.02	0.09	.858	1.02	0.09	.856
Minor urban (ref. main urban)	1.00	0.09	.988	0.99	0.09	.892	0.96	0.09	.615
Rural (ref. main urban)	0.88	0.08	.100	0.88	0.08	.098	0.85	0.08	.047
NZDep3–4 (ref. NZDep1–2)	1.16	0.09	.081	1.16	0.09	.091	1.18	0.09	.052
NZDep5–6 (ref. NZDep1–2)	1.21	0.09	.032	1.20	0.09	.041	1.24	0.09	.016
NZDep7–8 (ref. NZDep1–2)	1.34	0.09	.001	1.34	0.09	.001	1.42	0.09	<.001
NZDep9–10 (ref. NZDep1–2)	1.34	0.09	.001	1.32	0.09	.002	1.43	0.09	<.001
Māori (ref. European[b])	1.04	0.15	.795	1.36	0.07	<.001	1.19	0.09	.056
Pacific (ref. European[b])	0.99	0.12	.967	1.09	0.09	.321	0.94	0.10	.539
Asian (ref. European[b])	0.73	0.11	.003	0.86	0.09	.088	0.72	0.09	<.001
Other (ref. European[b])	0.86	0.21	.459	1.27	0.11	.032	0.84	0.17	.301
Māori/European (ref. European[b])	1.29	0.09	.003		-			-	
Pacific/European (ref. European[b])	1.16	0.13	.265		-			-	
Asian/European (ref. European[b])	1.08	0.17	.652		-			-	
Māori/Pacific (ref. European[b])	1.10	0.27	.715		-			-	
Māori/Pacific/European (ref. European[b])	1.92	0.20	.001		-			-	
2 groups NEI (ref. European[b])	1.36	0.10	.003		-			-	
≥3 groups NEI (ref. European[b])	1.89	0.13	<.001		-			-	
Can't choose one ethnic group (ref. European[b])		-			-		1.18	0.22	.450

Note. NEI = not elsewhere included.

[a]0 = no self-harm in the past 12 months; 1 = self-harm in the past 12 months.

[b]For the sole/combination and administrative-prioritisation models, the referent is *sole* European; for the self-prioritisation model, the referent is *self-prioritised* European.

Supplementary Table S6

Binary Logistic Regression Coefficients for Suicide Attempt[a] Using Non-Mutually Exclusive Ethnic Classification Methods

Variable	European model			Māori model			Pacific model			Asian model			Other ethnicities model		
	Exp(B)	*SE*	*p*	*Exp(B)*	*SE*	*p*	*Exp(B)*	*SE*	*p*	*Exp(B)*	*SE*	*p*	*Exp(B)*	*SE*	*p*
**Original total response models**															
Intercept	0.01	0.24	<.001	0.01	0.21	<.001	0.01	0.21	<.001	0.01	0.21	<.001	0.01	0.21	<.001
Female (ref. male)	2.87	0.13	<.001	2.91	0.13	<.001	2.81	0.13	<.001	2.88	0.13	<.001	2.87	0.13	<.001
Age 14 (ref. age ≤13 years)	1.56	0.16	.007	1.56	0.16	.007	1.56	0.16	.007	1.56	0.16	.007	1.56	0.16	.007
Age 15 (ref. age ≤13 years)	1.25	0.17	.204	1.27	0.17	.174	1.26	0.17	.188	1.25	0.17	.203	1.25	0.17	.193
Age 16 (ref. age ≤13 years)	1.31	0.18	.122	1.35	0.18	.087	1.33	0.18	.104	1.32	0.18	.121	1.34	0.18	.102
Age ≥17 (ref. age ≤13 years)	0.95	0.19	.811	1.00	0.19	.997	0.99	0.19	.942	0.96	0.19	.817	0.98	0.19	.900
Minor urban (ref. main urban)	1.01	0.17	.954	0.93	0.17	.669	1.15	0.17	.423	1.00	0.17	.977	1.00	0.17	.976
Rural (ref. main urban)	1.03	0.17	.859	0.96	0.17	.791	1.11	0.17	.540	1.02	0.17	.899	1.01	0.17	.935
NZDep3–4 (ref. NZDep1–2)	1.02	0.21	.919	1.03	0.21	.897	1.00	0.21	.992	1.03	0.21	.893	1.02	0.21	.911
NZDep5–6 (ref. NZDep1–2)	1.40	0.19	.084	1.39	0.19	.094	1.33	0.20	.148	1.42	0.19	.073	1.41	0.19	.074
NZDep7–8 (ref. NZDep1–2)	1.38	0.20	.102	1.33	0.20	.149	1.25	0.20	.256	1.41	0.20	.080	1.39	0.20	.094
NZDep9–10 (ref. NZDep1–2)	2.55	0.18	<.001	2.50	0.17	<.001	1.98	0.18	<.001	2.69	0.17	<.001	2.63	0.17	<.001
European (ref. non-European)	0.87	0.13	.256		-			-			-			-	
Māori (ref. non-Māori)		-		1.49	0.12	.001		-			-			-	
Pacific (ref. non-Pacific)		-			-		1.89	0.14	<.001		-			-	
Asian (ref. non-Asian)		-			-			-		1.11	0.15	.490		-	
Other (ref. non-Other)		-			-			-			-		1.62	0.15	.002
**Modified total response models**															
Intercept				0.01	0.27	<.001	0.01	0.29	<.001	0.01	0.31	<.001	0.01	0.31	<.001
Female (ref. male)				2.87	0.17	<.001	3.23	0.17	<.001	2.59	0.19	<.001	3.08	0.20	<.001
Age 14 (ref. age ≤13 years)				1.33	0.21	.172	1.77	0.21	.008	1.54	0.27	.104	1.35	0.26	.257
Age 15 (ref. age ≤13 years)				1.12	0.22	.616	1.29	0.23	.264	1.39	0.27	.222	1.31	0.27	.311
Age 16 (ref. age ≤13 years)				1.28	0.23	.283	1.66	0.23	.025	1.51	0.27	.129	1.97	0.26	.008
Age ≥17 (ref. age ≤13 years)				0.95	0.25	.849	0.96	0.26	.876	1.26	0.28	.406	1.10	0.30	.742
Minor urban (ref. main urban)				1.02	0.20	.937	1.25	0.23	.331	0.80	0.29	.441	1.03	0.26	.920
Rural (ref. main urban)		-[b]		1.11	0.19	.565	1.35	0.23	.184	1.48	0.22	.079	1.45	0.21	.079
NZDep3–4 (ref. NZDep1–2)				1.05	0.25	.846	0.79	0.29	.413	0.91	0.27	.732	0.78	0.27	.351
NZDep5–6 (ref. NZDep1–2)				1.43	0.23	.128	1.54	0.25	.088	1.79	0.24	.016	1.42	0.24	.152
NZDep7–8 (ref. NZDep1–2)				1.34	0.24	.225	1.25	0.27	.410	1.30	0.28	.339	1.19	0.27	.515
NZDep9–10 (ref. NZDep1–2)				1.59	0.24	.057	1.83	0.26	.018	2.66	0.26	<.001	2.05	0.26	.006
Māori (ref. sole European)				2.31	0.16	<.001		-			-			-	
Pacific (ref. sole European)					-		2.74	0.18	<.001		-			-	
Asian (ref. sole European)					-			-		1.69	0.19	.005		-	
Other (ref. sole European)					-			-			-		2.52	0.19	<.001

[a]0 = no suicide attempt in the past 12 months; 1 = suicide attempt in the past 12 months.

[b]Model not applicable as the reference group for modified total response is sole European.

Supplementary Table S7

Binary Logistic Regression Coefficients for Suicide Attempt[a] Using Mutually Exclusive Ethnic Classification Methods

Variable	Sole/combination model			Administrative-prioritisation model			Self-prioritisation model		
	Exp(B)	*SE*	*p*	*Exp(B)*	*SE*	*p*	*Exp(B)*	*SE*	*p*
Intercept	0.01	0.22	<.001	0.01	0.22	<.001	0.01	0.21	<.001
Female (ref. male)	2.85	0.13	<.001	2.85	0.13	<.001	2.84	0.13	<.001
Age 14 (ref. age ≤13 years)	1.56	0.16	.007	1.55	0.16	.008	1.53	0.16	.009
Age 15 (ref. age ≤13 years)	1.30	0.17	.134	1.27	0.17	.162	1.24	0.17	.208
Age 16 (ref. age ≤13 years)	1.41	0.18	.056	1.37	0.18	.074	1.33	0.18	.111
Age ≥17 (ref. age ≤13 years)	1.06	0.20	.784	1.02	0.20	.917	0.98	0.20	.916
Minor urban (ref. main urban)	1.16	0.17	.396	1.13	0.17	.482	1.09	0.17	.610
Rural (ref. main urban)	1.12	0.18	.528	1.10	0.18	.585	1.07	0.18	.680
NZDep3–4 (ref. NZDep1–2)	0.97	0.21	.888	0.96	0.21	.854	0.98	0.21	.941
NZDep5–6 (ref. NZDep1–2)	1.25	0.20	.250	1.23	0.20	.287	1.30	0.20	.183
NZDep7–8 (ref. NZDep1–2)	1.09	0.20	.666	1.08	0.20	.716	1.17	0.20	.443
NZDep9–10 (ref. NZDep1–2)	1.71	0.19	.004	1.66	0.19	.007	1.81	0.19	.002
Māori (ref. European[b])	1.83	0.29	.037	2.26	0.15	<.001	1.90	0.17	<.001
Pacific (ref. European[b])	2.41	0.22	<.001	2.58	0.18	<.001	2.04	0.17	<.001
Asian (ref. European[b])	1.10	0.25	.693	1.43	0.20	.076	1.10	0.20	.639
Other (ref. European[b])	1.50	0.43	.347	2.00	0.23	.003	2.12	0.28	.008
Māori/European (ref. European[b])	1.83	0.18	.001		-			-	
Pacific/European (ref. European[b])	2.63	0.23	<.001		-			-	
Asian/European (ref. European[b])	2.33	0.32	.008		-			-	
Māori/Pacific (ref. European[b])	3.29	0.40	.003		-			-	
Māori/Pacific/European (ref. European[b])	3.38	0.34	<.001		-			-	
2 groups NEI (ref. European[b])	2.44	0.20	<.001		-			-	
≥3 groups NEI (ref. European[b])	3.44	0.23	<.001		-			-	
Can’t choose one ethnic group (ref. European[b])		-			-		1.66	0.43	.238

Note. NEI = not elsewhere included.

[a]0 = no suicide attempt in the past 12 months; 1 = suicide attempt in the past 12 months.

[b]For the sole/combination and administrative-prioritisation models, the referent is *sole* European; for the self-prioritisation model, the referent is *self-prioritised* European.
